# Supplementary material for: Proteomic analyses identify ARH3 as a serine mono-ADP-ribosylhydrolase
Source: Nat Commun. 2017 Dec 12;8:2055. doi: 10.1038/s41467-017-02253-1 (PMC5727137; doi:10.1038/s41467-017-02253-1)
Supplement: Supplementary file 1 — Supplementary Information [file 41467_2017_2253_MOESM1_ESM.pdf]

Supplementary Information

Supplementary Figure 1

a

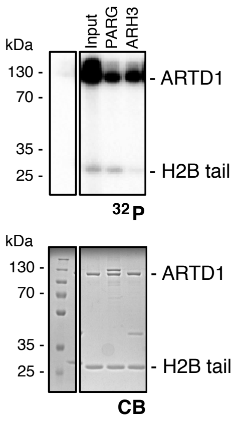

b

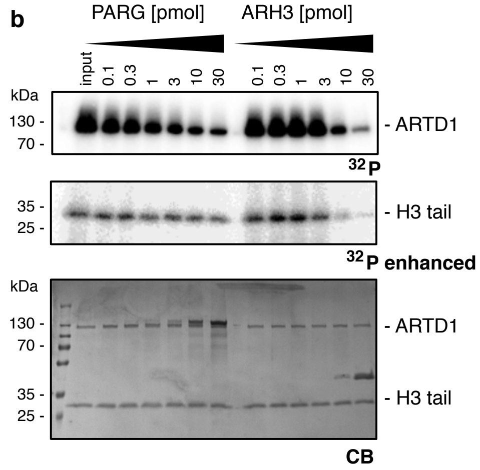

c

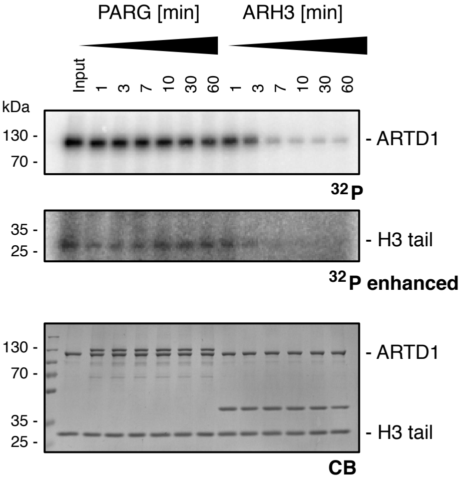

d

Residues	Potential function	introduced mutation
E41<br>D77	Holding water molecule in place	E41Q*<br>D77N*
G115		G115D
D314<br>T317<br>E41	Holding Mg^{2+} ion in place	D314A*<br>T317A*<br>E41A*
S148<br>H182<br>Y149	Binding of substrate (i.e. adenine and phosphate)	S148A*<br>H182A*<br>Y149A*

e

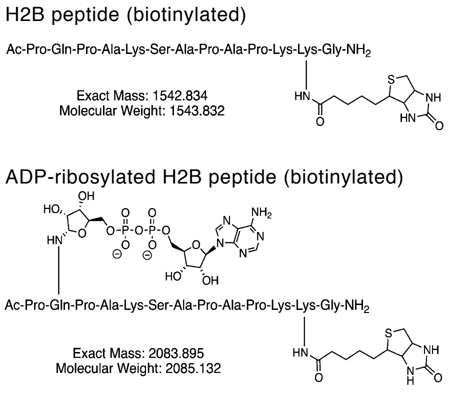

f

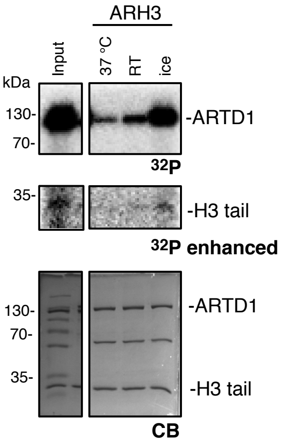

Supplementary Figure 1: (**a**) Recombinant H2B histone tail was *in vitro* ADP-ribosylated using recombinant ARTD1 in the presence of ^{32}P-labeled NAD^{+}. Equal fractions were left untreated (Input) or were treated with PARG or ARH3. Above: radioactivity exposure, below: Coomassie Blue-stained poly-acrylamide gel. (**b**) Dose response of ARH3 and PARG on auto-modified ARTD1 and transmodified H3 histone tail (numbers indicate pmol of PARG and ARH3 used for demodification). (**c**) Time course of PARG and ARH3 activity on auto-modified ARTD1 and transmodified H3 histone tail (numbers indicate duration of the PARG and ARH3 demodification reaction in min). (**d**) Scheme representing the cloned and purified ARH3 mutants that were used for binding and demodification assays. (**e**) Schematic structure of the biotinylated H2B peptides with and without ADPr-modification. (**f**) Recombinant H3 histone tail was *in vitro* ADP-ribosylated using recombinant ARTD1 in the presence of ^{32}P-labeled NAD^{+}. Equal fractions were left untreated (Input) or were treated with ARH3 at different temperatures. Above: radioactivity exposure, below: Coomassie Blue-stained poly-acrylamide gel.

Supplementary Figure 2

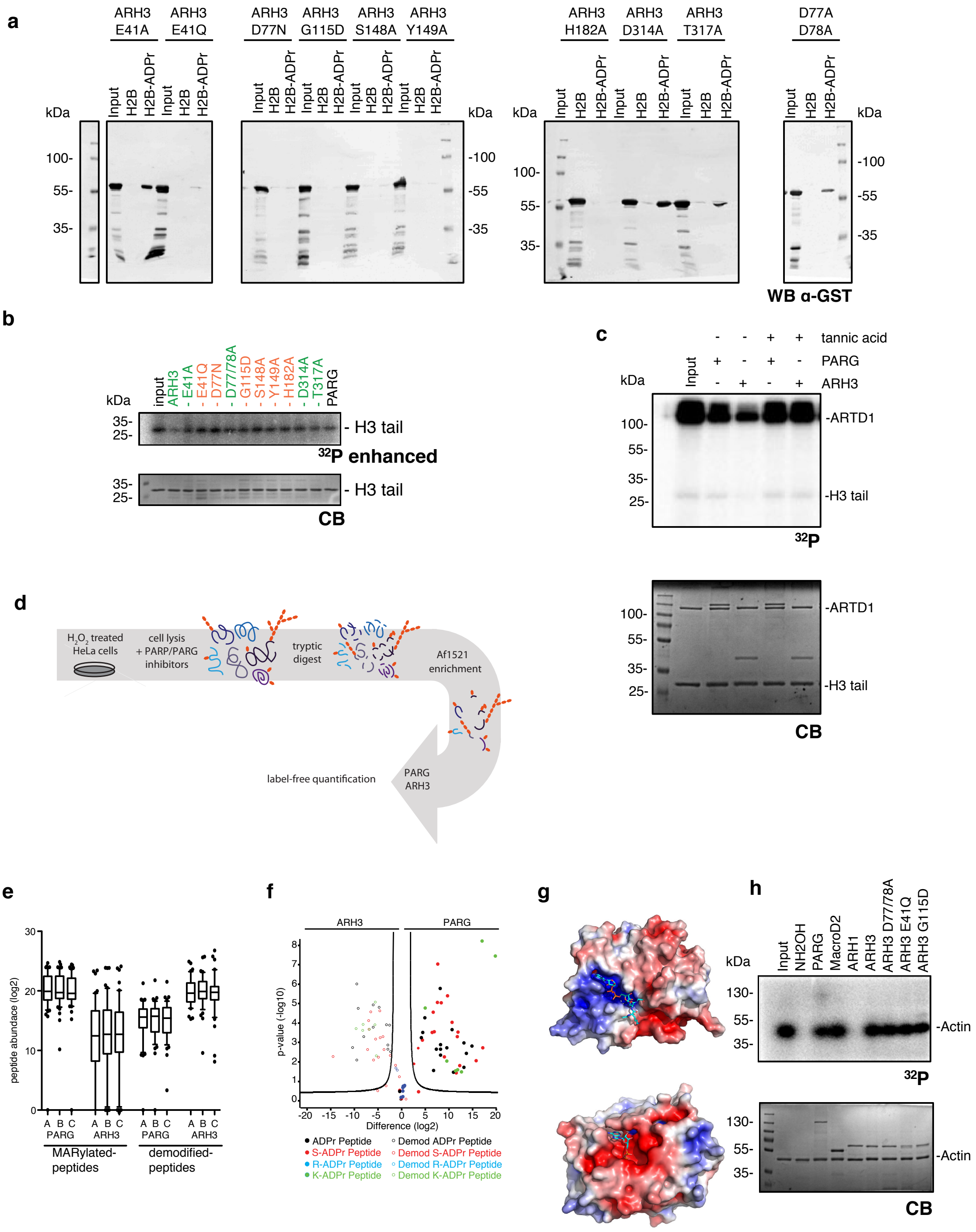

Supplementary Figure 2: (**a**) Western blot of pull-downs of GST-ARH3 mutants using the biotinylated peptides with (H2B-ADPr) and without (H2B) modification for assessment of binding capacity of ARH3 mutants to MARylated peptides. The blots were probed with an anti-GST antibody. (**b**) Recombinant H3 histone tail was *in vitro* ADP-ribosylated using recombinant ARTD1 in the presence of ^{32}P-labeled NAD$^+$. Equal fractions were left untreated (Input) or were treated with PARG, wildtype and mutant ARH3. Above: radioactivity exposure, below: Coomassie Blue-stained poly-acrylamide gel. (**c**) Recombinant H3 histone tail was *in vitro* ADP-ribosylated using recombinant ARTD1 in the presence of ^{32}P-labeled NAD$^+$. Equal fractions were left untreated (Input) or were treated with PARG and ARH3 without or in presence of tannic acid. Above: radioactivity exposure, below: Coomassie Blue-stained poly-acrylamide gel. (**d**) Schematic representation of the sample preparation for the LFQ MS experiment. For details see Materials and Methods. (**e**) Normalized abundance of individual ADP-ribosylated or demodified peptide species from three peptide preparations (A, B, C) that were treated with either ARH3 or PARG. (**f**) Volcano plot as in Figure 2c using high scoring EThcD and HCD fragmentation spectra. 'Demodified peptides' are shown as open circles and 'ADP-ribosylated peptides' as filled circles. Confident ADP-ribosylation sites identified by EThcD and HCD are annotated and color coded in red as S-ADPr, in blue as R-ADPr sites and in green as K-ADPr sites. ADP-ribosylated peptides with uncertain ADP-ribosylation site localization are shown in black. The black hyperbolic line represents a permutation-based False Discovery Rate (FDR) of 5% and a minimal fold change of 2. (**g**) Comparison of ARH3 (PDB code: 2FP0, with a modeled ADP-ribosylated serine within an acetyl lysine-serine-glycine (KSG) tripeptide in the binding site) and ARH1 (PDB code: 3HFW, with adenosine diphosphate). Protein surfaces are colored according to electrostatic potential (on a scale of -5 to 5 kT/e). (**h**) Actin was *in vitro* ADP-ribosylated with the bacterial toxin CDTa in the presence of ^{32}P-labeled NAD$^+$. Equal fractions were left untreated (Input) or were treated with hydroxylamine, PARG, MACROD2, ARH1, wildtype and mutant ARH3. Above: radioactivity exposure, below: Coomassie Blue-stained poly-acrylamide gel.

Supplementary Figure 3

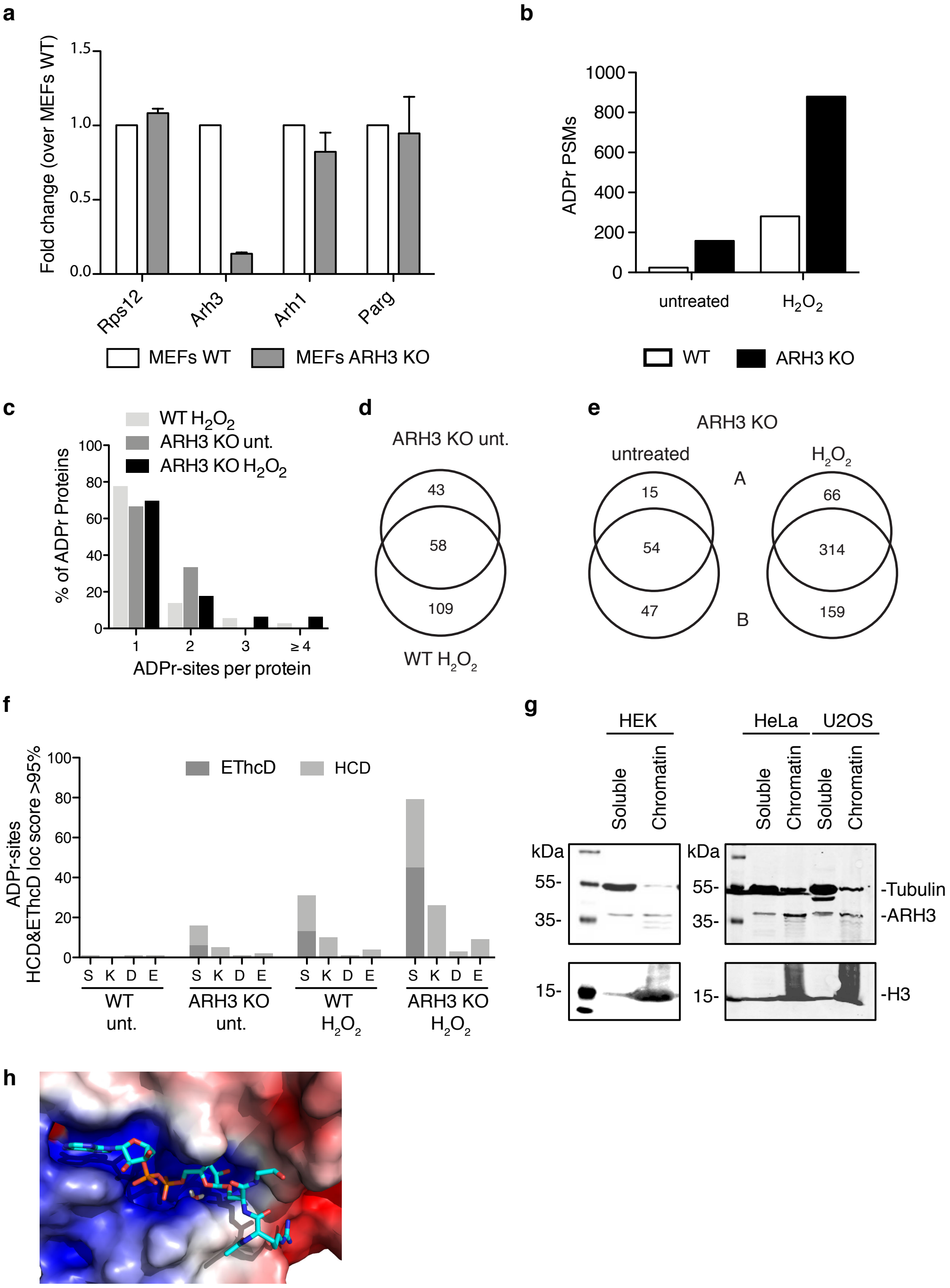

Supplementary Figure 3: (**a**) mRNA levels of *Rps12* (house keeping gene), *Arh3*, *Arh1* and *Parg* in untreated ARH3 WT and KO MEF cells assessed by qRT-PCR. Data represent means ± SEM for *n* = 3 independent experiments. (**b**) ADP-ribosylated peptide spectra matches of WT and ARH3 KO cells in untreated and hydrogen peroxide-treated conditions. (**c**) Number of ADPr sites per protein. (**d**) Venn diagrams of unique ADP-ribosylated peptides from H_2O_2-treated WT and untreated ARH3 KO MEFs as in Fig. 3a. (**e**) Venn diagrams of unique ADP-ribosylated peptides identified in biological replicates (samples A and B). (**f**) Number of unique ADP-ribose acceptor amino acids localized with EThcD and HCD. (**g**) Chromatin extraction of HEK, HeLa and U2OS cells followed by western blot analysis with α-tubulin (predominantly found in the soluble fraction), α-H3 (marking the chromatin-bound fraction) and α-ARH3 (detected in both fractions). (**h**) Energy minimized binding mode of an acetyl-RSG peptide with ADPr-Ser modified. The surface of ARH3 (including the binding site magnesium ions) is colored according to electrostatic potential. The positively charged amino group of the R side chain and backbone amide groups point towards the region of the surface with negative potential.

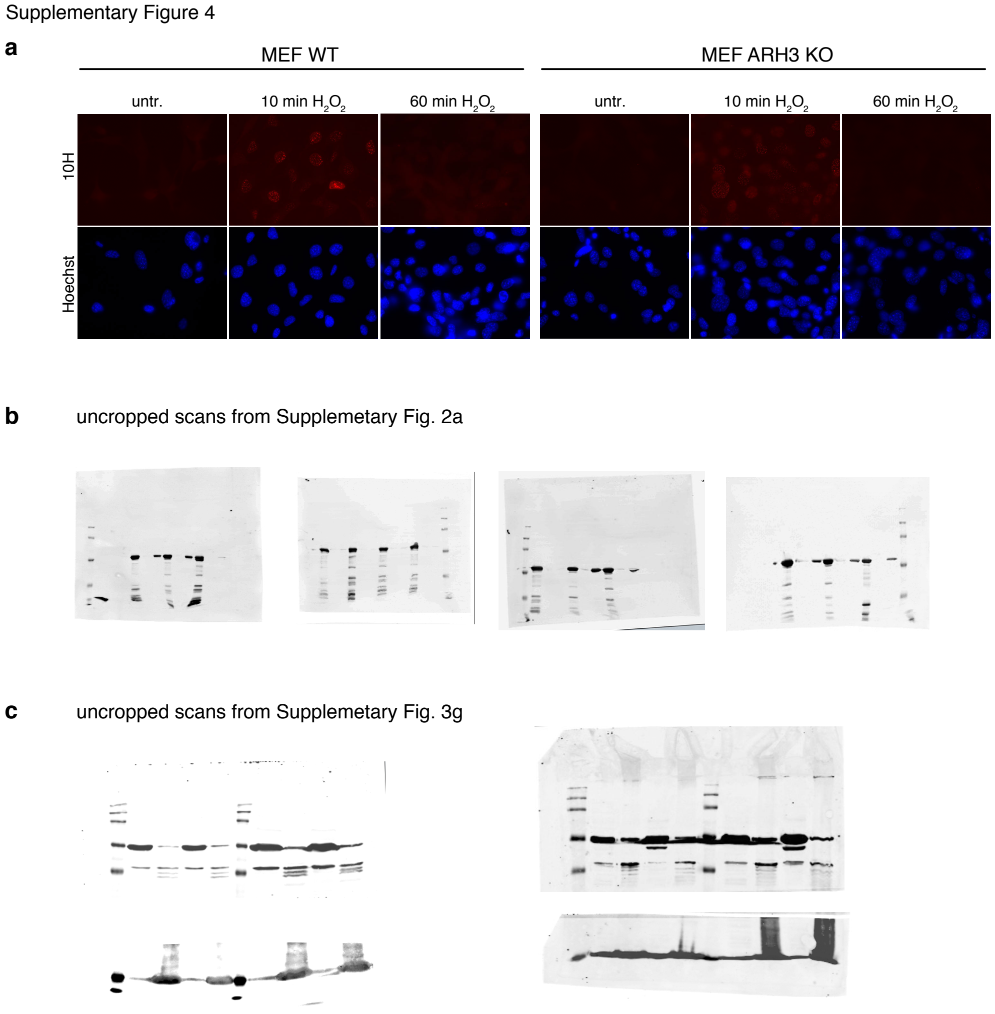

Supplementary Figure 4: (a) Immunofluorescence pictures showing PAR-staining in WT and ARH3 KO MEF cells. **(b)** Full blots for Supplementary Fig. 2a. **(c)** Full blots for Supplementary Fig. 3g**.**
