# Supplementary material for: Proteomic analyses identify ARH3 as a serine mono-ADP-ribosylhydrolase
Source: Nat Commun. 2017 Dec 12;8:2055. doi: 10.1038/s41467-017-02253-1 (PMC5727137; doi:10.1038/s41467-017-02253-1)
Supplement: Supplementary file 4 — Descriptions of Additional Supplementary Files [file 41467_2017_2253_MOESM4_ESM.pdf]

Descriptions of Additional Supplementary Files

File Name: Supplementary Dataset 1
Description: List of identified ADP-ribosylated peptides and their non-modified counterparts after treatment with ARH3 or PARG, quantified by Progenesis.

File Name: Supplementary Dataset 2
Description: List of identified ADP-ribosylated peptides in WT and ARH3 KO MEFs, untreated and H2O2 treated.
